# Roles and functions of IAV proteins in host immune evasion

**DOI:** 10.3389/fimmu.2023.1323560

**Published:** 2023-12-13

**Authors:** Farooq Rashid, Zhixun Xie, Meng Li, Zhiqin Xie, Sisi Luo, Liji Xie

**Affiliations:** ^1^ Department of Biotechnology, Guangxi Veterinary Research Institute, Nanning, China; ^2^ Guangxi Key Laboratory of Veterinary Biotechnology, Nanning, China; ^3^ Key Laboratory of China (Guangxi)-ASEAN Cross-border Animal Disease Prevention and Control, Ministry of Agriculture and Rural Affairs of China, Nanning, China

**Keywords:** IAVs, IAV proteins, immune evasion, host immune system, IFNs

## Abstract

Influenza A viruses (IAVs) evade the immune system of the host by several regulatory mechanisms. Their genomes consist of eight single-stranded segments, including nonstructural proteins (NS), basic polymerase 1 (PB1), basic polymerase 2 (PB2), hemagglutinin (HA), acidic polymerase (PA), matrix (M), neuraminidase (NA), and nucleoprotein (NP). Some of these proteins are known to suppress host immune responses. In this review, we discuss the roles, functions and underlying strategies adopted by IAV proteins to escape the host immune system by targeting different proteins in the interferon (IFN) signaling pathway, such as tripartite motif containing 25 (TRIM25), inhibitor of nuclear factor κB kinase (IKK), mitochondrial antiviral signaling protein (MAVS), Janus kinase 1 (JAK1), type I interferon receptor (IFNAR1), interferon regulatory factor 3 (IRF3), IRF7, and nuclear factor-κB (NF-κB). To date, the IAV proteins NS1, NS2, PB1, PB1-F2, PB2, HA, and PA have been well studied in terms of their roles in evading the host immune system. However, the detailed mechanisms of NS3, PB1-N40, PA-N155, PA-N182, PA-X, M42, NA, and NP have not been well studied with respect to their roles in immune evasion. Moreover, we also highlight the future perspectives of research on IAV proteins.

## Introduction

The RNA genome of influenza viruses is segmented, negative sense, and belongs to the family *Orthomyxoviridae*. Influenza viruses are divided into four genera, A, B, C, and D (1). Influenza viruses A and B cause all seasonal epidemics, whereas influenza viruses C and D cause mild disease in specific hosts (2, 3). Their genomes consist of eight single-stranded segments, including nonstructural proteins (NS), basic polymerase 1 (PB1), basic polymerase 2 (PB2), hemagglutinin (HA), acidic polymerase (PA), matrix (M), neuraminidase (NA), and nucleoprotein (NP) (4). The NS proteins encode at least 14 proteins and have a wide range of functions, including antagonism of host immune responses (5). PB1, PB2, and PA are polymerase components and are responsible for IAV genome replication and transcription (6–8). The HA protein recognizes host cells and mediates IAV entry into host cells (9, 10), M provides structural support to virions, NA is a sialidase and mediates virion release from cells, and NP is an RNP component and is responsible for RNA encapsidation.

Influenza A virus (IAV) is a serious risk to both animals and humans because it causes annual seasonal epidemics and has resulted in severe pandemic outbreaks (11, 12). A novel IAV strain can be generated after the coinfection of a host with two or more different viruses followed by a reassortment process, after which, the strain expresses new combinations of HA and NA subtypes; therefore, IAVs are subtyped by the antigenic characteristics of their HA and NA glycoproteins (13). To date, all human pandemics have occurred due to genetic reassortment between human, swine, and avian influenza viruses (AIVs) (14). The fast and frequent mutations and recombination process of influenza viruses have resulted in highly pathogenic strains of IAVs, e.g., H5N1, H5N6, H5N8, and H7N9 (15). To date, eighteen different HA subtypes (H1-H18) and eleven NA subtypes (N1-N11) have been reported, and the most recently identified H17N10 and H18N11 subtypes have been discovered in New World bats and have not been found in humans or any avian species (16, 17). The most lethal example of a new influenza virus subtype was recorded in 1918 when the H1N1 (H1 subtype, N1 subtype) virus (Spanish flu) caused the death of approximately 100 million people (18). This subtype was replaced by H2N2 (H2 subtype, N2 subtype) virus (Asian flu) in 1957, which had novel H2, the N2, and PB1 genes derived from a Eurasian avian virus source (19). In 1968, the H3N2 (H3 subtype, N2 subtype retained) virus (Hong Kong flu) emerged, and in 1977, a small but notorious H1N1 virus of the 1950s reemerged in Russia (Russian flu). A novel pandemic H1N1 virus (swine flu) emerged in 2009 in Mexico, which spread around the globe and replaced the previous H1N1 viruses that circulated for approximately thirty years until 2009 (14, 18, 19).

**Figure 1:**
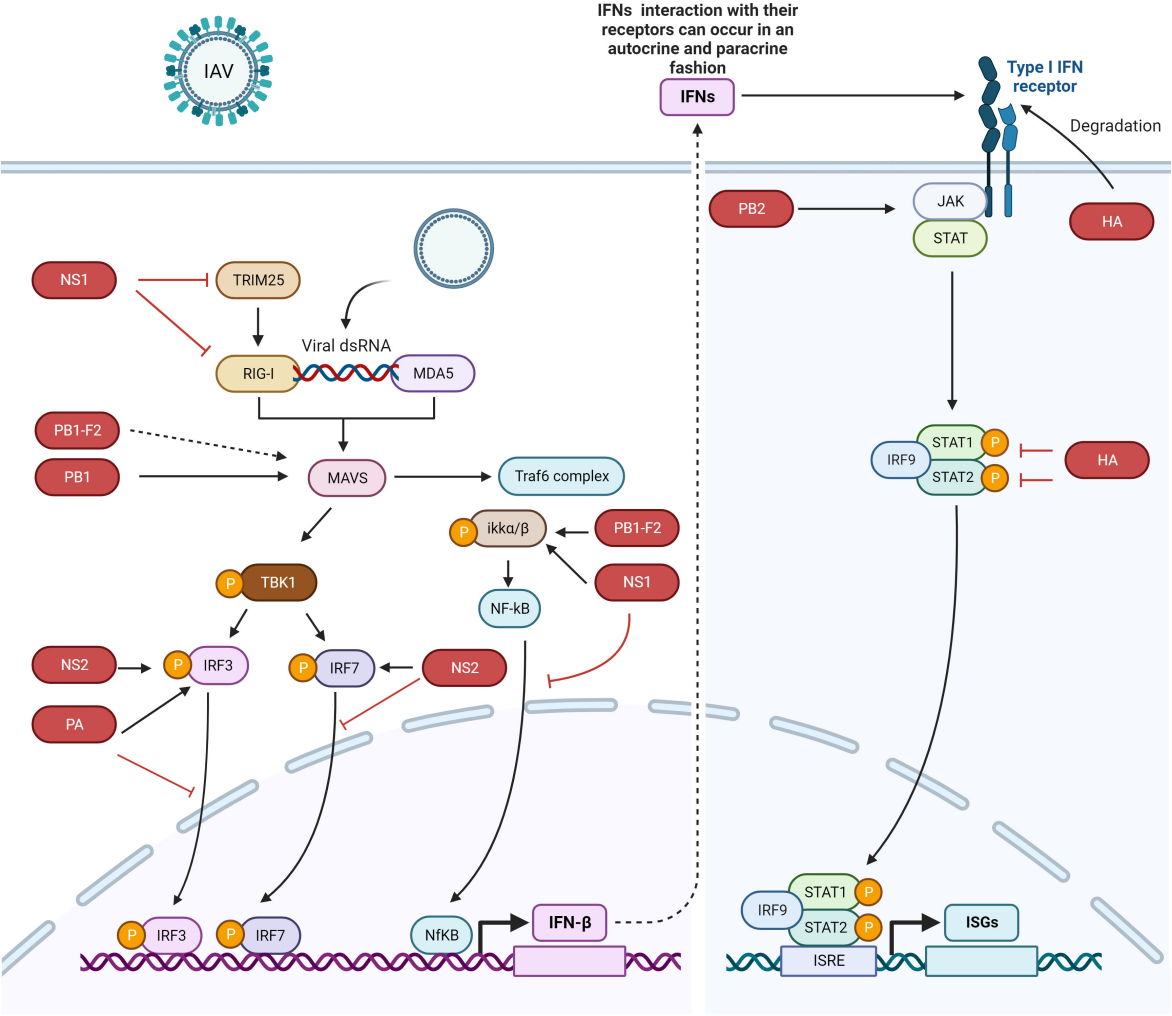
IFN signaling pathway activation and evasion by IAV proteins. The viral genome is recognized by RIG-I and MDA5. IRF3, IRF7, and NF-κB trigger the production of IFNs. IFNs are secreted to induce the JAK/STAT pathway, which express ISGs. Different IAV proteins antagonize different proteins in the host innate immune signaling pathway. NS1 binds with IKK, RIG-I, and TRIM25 to escape the host immune system, NS2 interacts with IRF7 to suppress its nuclear translocation, the PB1 protein interacts with and degrades MAVS, and PB1-F2 probably interacts with MAVS to inhibit IFN production (dotted lines indicate that the interaction between these two proteins has not yet been confirmed). PB1-F2 also binds with IKKβ to suppress NF-κB signaling. The HA protein degrades IFNAR1, and PA binds with and inhibits the nuclear translocation of IRF3. The dotted arrow indicates that the interaction between the proteins has not yet been confirmed. The figure was created with BioRender.com.

Interferons (IFNs) are the first line of host defense against invading viruses. The innate immune response is activated by the recognition of viral pathogen-associated molecular patterns (PAMPs) by retinoic acid-inducible gene-I (RIG-I) and melanoma differentiation-associated gene 5 (MDA5) (12) (**Figure 1**). After activation, RIG-I and MDA5 interact with mitochondrial antiviral signaling protein (MAVS) and eventually activate MAVS. After MAVS activation, downstream signaling proteins are recruited to the mitochondria, leading to the activation of an inhibitor of NF-κB kinase (IKKϵ) and TANK-binding kinase (TBK1) and finally resulting in the phosphorylation of the transcription factors interferon regulatory factor 3 (IRF3) and IRF7. Phosphorylated IRF3 and IRF7, as well as activated nuclear factor kappa-light-chain-enhancer of activated B cells (NF-κB), trigger the expression of interferons (IFNs) and cytokines (20) and thus create an antiviral state to counteract the pathogenesis of IAVs (12, 21–24).

TLR3, TLR7, TLR 8, and TLR 9 recognize PAMPs derived from several viruses, including IAVs (25–27). TLR3 recognizes dsRNA in endosomes, while TLR7 recognizes the ssRNA of influenza viruses and is activated after interaction (20, 28, 29). Activated TLR7 leads to the activation of IFN-I or proinflammatory cytokines (20), while TLR3 activation leads to IFN-β activation (30).

The expressed IFN-I interacts with IFN-α/β receptors (IFNAR), and IFN-III interacts with IFNL receptors (IFNLR) in an autocrine or paracrine manner (31). Secretion of IFN-I and III leads to the activation of Janus kinase 1 (JAK1) and tyrosine kinase 2 (Tyk2), followed by the phosphorylation of signal transducer and activator of transcription 1 and 2 (STAT1 and STAT2) (32, 33). Phosphorylated STAT1 and STAT2 form heterodimers and interact with IRF9 to form the IFN-stimulated growth factor 3 (ISGF3) complex. This complex is translocated into the nucleus, where at the ISG promoter, it interacts with IFN-stimulated response elements (ISREs) to transcribe its downstream genes, which counteract viral infection (**Figure 1**).

However, IAVs have developed mechanisms to counteract IFNs and cultivate successful infection. The different proteins of IAVs act in different manners by targeting different steps of the IFN signaling pathway. To date, several IAV proteins have been identified that evade the host immune system through various strategies. The following sections elaborate on how the different IAV proteins adopt different strategies to target different proteins of the IFN signaling pathway and establish successful infection.

## NS1 inhibits IFN-I activation by multiple mechanisms

NS1, a nonstructural protein of IAV, is considered the most potent inhibitor of the host innate immune system during viral replication (1, 34). The smallest RNA segment of IAV encodes the NS1 protein in conjunction with NEP, which is an NS mRNA splice variant (35). NS1 promotes IAV replication by using its functional domains that interact with both viral and cellular proteins. The RNA-binding domain (RBD) of NS1 at the amino terminal end interacts with dsRNA molecules to block the induction of RLR sensors such as OASL and PKR (36). This mechanism of host immune response inhibition by NS1 was considered the main strategy; however, later, several other interactions involved in these mechanisms were later found, and these interactions are discussed below (37).

Tripartite motif containing 25 (TRIM25) is a ubiquitin ligase that mediates lysine 63-linked ubiquitination of the N-terminal CARD domain of RIG-I to trigger IFN-I production (38). NS1 of IAV interacts with TRIM25 and thus blocks TRIM25-mediated RIG-I signaling. A domain in NS1 that contains residues E96/97 is responsible for interacting with the TRIM25 coil-coil domain to inhibit RIG-I CARD ubiquitination. IAV with mutations in the NS1 domain at residues E96/97, i.e., E96A/E97A, was unable to block IFN responses mediated by TRIM25, and virulence in mice was lost (38). In 2012, Rajsbaum et al. further studied the mechanisms of host tropism and viral adaptation, and for this purpose, they studied the ability of NS1 from avian (HK156), human (CaI04), mouse (PR8) and swine adapted (SwTx98) influenza viruses to interact with TRIM25 encoded by avian and mammalian species (39). Coimmunoprecipitation experiments indicated that TRIM25 in humans interacts with all NS1 proteins under study; however, TRIM25 in chickens interacts preferentially with the avian virus NS1. In addition, none of the NS1 proteins interact with TRIM25 in mice. Furthermore, the researchers determined the effect of NS1 and TRIM25 on RIG-I ubiquitination in mouse cells and found that NS1 inhibited the ubiquitination of full-length mouse RIG-I in a mouse TRIM25-independent manner. They further wanted to know if Riplet interacts with NS1, as Riplet is already known to ubiquitinate RIG-I. NS1 binds with Riplet from mice in murine cells and blocks the function of Riplet to induce IFN-β. Only the human NS1 protein was able to bind to human Riplet to suppress RIG-I ubiquitination; however, the NS1 of avian or swine viruses was unable to bind Riplet from humans. Therefore, influenza NS1 targets Riplet and TRIM25 in a species-specific manner to suppress RIG-I ubiquitination and IFN production (39). A previous study (40) demonstrated that the NS1 protein binds with RIG-I and therefore suppresses the RIG-I-mediated activation of IFN-β. Moreover, they found that the NS1 of IAV binds with RIG-I and inhibits IRF3 activation and its nuclear translocation (40). This study was further elaborated upon by Jureka et al. (41), who delineated through biophysical and structural evidence that the binding between RIG-I and the IAV NS1 protein is direct and that the RNA binding domain (RBD) of NS1 and the second caspase activation and recruitment domain of RIG-I are responsible for this interaction (41).

NS1 also inhibits IFN activation by interacting with the protein kinase PKR (42, 43). Additionally, NS1 blocks host antiviral activity by interacting with the 30-kDa subunit of the cleavage and polyadenylation specificity factor (CPSF30) to process cellular mRNA (44, 45). Furthermore, NS1 inhibits the NK-κB pathway by targeting the inhibitor of κB kinase (IKK), and therefore, the expression of antiviral genes is blocked (**Figure 1**, **Table 1**) (46). The phosphorylation of IKKα/IKKβ is affected in both compartments of cells, i.e., the cytoplasm and nucleus when interacting with NS1. Furthermore, NF-κB translocates into the nucleus, and the expression of its downstream target genes is also inhibited. The phosphorylation of histone H3 Ser 10, which is mediated by IKK, is also impaired by NS1 (46).

**Table 1:** IAV proteins that interfere with IFN induction and signaling.

Protein	Mechanism	Experimental approach	Cellular Model	References
IFN production inhibition				
**NS1**	Interacts with TRIM25 and Riplet to block RIG-I activation, interacts with RIG-I to inhibit nuclear translocation of IRF3, interacts with IKK to inhibit NF-κB nuclear translocation and suppress the expression of NF-κB target genes	Coimmunoprecipitation, Western blotting, immunofluorescence assay, mouse experiments, biophysical approaches	HEK293T cells, A549 cells, Hepa 1.6 (Mouse), Vero, L929 cells	(38–41, 46)
**NS2**	Interacts with and blocks nuclear translocation of IRF7 to inhibit IFN production	Coimmunoprecipitation, Western blotting, immunofluorescence assay	HEK293 cells, A549 cells	(12)
**PB1**	Interacts with and degrades MAVS	Coimmunoprecipitation, Western blotting	HEK293, A549 cells, HeLa cells	(47)
**PB1-F2**	Inhibits IFN production at MAVS level, PB1-F2 interacts with IKKβ and inhibits NF-κB signaling	Luciferase assay, immunofluorescence assay, Western blotting, coimmunoprecipitation, yeast two-hybrid assays	293T cells, African green monkey kidney Vero cells	(48, 49)
**PA**	Interacts with and blocks nuclear translocation of IRF3 to inhibit IFN production	Coimmunoprecipitation, Western blotting, immunofluorescence assay	HEK293T cells, A549 cells	(50)
IFN signaling inhibition				
**PB2**	Interacts with JAK1 and inhibits JAK1/STAT signaling	Luciferase assay, immunofluorescence assay, coimmunoprecipitation, mouse experiments	HEK293T cells, DF-1 cells, A549 cells	(51)
**HA**	Degrades IFNAR1 and IFNGR1, decreases STAT1/STAT2 phosphorylation	Western blotting, immunoprecipitation, ubiquitination assays, luciferase assay	HEK293 cells, A549 cells, African green monkey kidney Vero cells	(52, 53)

Another mechanism that involves the obstruction of the host innate immune response is NS1 induction of the degradation of sphingosine 1-phosphate lyase (SPL) (54). SPL was previously shown to enhance IKKϵ-mediated IFN-I responses (55). IAV infections or NS1 expression caused ubiquitination and downregulation of SPL expression; however, IAV deficient in NS1 failed to downregulate SPL expression (54).

The Hippo pathway regulates homeostasis and organ development (56, 57). The important proteins of this pathway in mammals are the kinase cascade involving mammalian STE20-like protein kinase 1 (MST1) and mammalian STE20-like protein kinase 2 (MST2); the large tumor suppressor 1 (LATS1) and large tumor suppressor 2 (LATS2); the adaptor proteins Salvador homolog 1 (SAV1) for MST1/2 and MOB kinase activators (MOB1A/MOB1B) for LATS1/2; downstream effectors Yes-associated protein (YAP) and its analog protein, transcriptional coactivator with PDZ-binding motif (TAZ); and TEA domain transcription factors (TEADs) (58–62). Once this pathway is activated, a series of phosphorylation events start via MST and LATS kinases, and finally, YAP/TAZ are phosphorylated. Phosphorylated YAP/TAZ is polyubiquitinated and degraded in the proteasome (63, 64). Reports have shown that the Hippo pathway also regulates the innate immune pathway (65, 66).

Another study demonstrated the relationship between the Hippo pathway and IAV infection in A549 cells (67). The YAP/TAZ effectors of the Hippo pathway were dephosphorylated, their expression was upregulated, and they were translocated into the nucleus after IAV infection. Furthermore, in this study, the researchers investigated whether the NS1 protein was responsible for the transcriptional activities of YAP/TAZ via the physical interaction between NS1 and YAP/TAZ. This study further dissected how YAP/TAZ suppress IAV-induced innate immune responses. Knockdown of YAP/TAZ induced the activation of proinflammatory and antiviral factors such as CXCL8, IFNB1, and IRF7, which are triggered by virus infection. Similarly, YAP overexpression decreased the activation of IFNB promoter activity, and the TAD domain at the C-terminus of YAP was indispensable for innate immune signaling. Furthermore, overexpression of YAP suppressed TLR3 expression, and deletion of the C-terminal TAD domain of YAP abolished TLR3 suppression. YAP binds to TEAD binding sites on the TLR3 promoter region. Furthermore, elimination of acetylated histone H3 occupancy in the TLR3 promoter led to its transcriptional silencing, and treatment with the histone deacetylase (HDAC) inhibitor trichostatin A reversed TLR3 expression inhibition mediated by YAP/TAZ. These results delineated the novel mechanism of innate immune system regulation by IAV, where YAP/TAZ inhibits TL3-mediated innate immunity (67).

NS1 of IAV is thus a multifunctional virulence factor, as it regulates the host machinery to facilitate viral replication. NS1 is under constant evolutionary pressure due to its high mutation rate and its infection of and replication in different hosts.

## NS2 interacts with IRF7 and suppresses its nuclear translocation to inhibit IFN-I production

H1N1-NS2 significantly inhibits the production of IFN-β, IFN-stimulated gene 56 (ISG56), and IFN-induced protein with tetratricopeptide repeats 2 (IFIT2) (12). NS2 colocalized with IRF3 and IRF7 and even physically interacted with these two proteins. The same study also found that NS2 significantly suppressed the dimerization of IRF7 and its nuclear translocation (**Figure 1**, **Table 1**). Furthermore, the indispensable domain in NS2 was determined based on its interaction with IRF7. Amino acid residues 1-53 in the N-terminal domain were found to be responsible for interacting with IRF7, while amino acid residues 54-121 in the C-terminal domain did not interact with IRF7; hence, amino acid residues 1-53 in the N-terminal domain of NS2 are responsible for inhibiting IFN-I production (12).

## PB1 interacts with and degrades MAVS to suppress the innate immune response

IAV polymerase plays a role in regulating innate immune responses in the host. PB1 suppresses the Sendai virus (SeV)- or poly (I:C)-triggered activation of the IFN-β promoter; however, it does not inhibit STAT1 activation induced by IFN-β. Overexpression of PB1 inhibited Sev- or poly (I:C)-induced transcription of the following genes: IFNB1, IFN-stimulated gene 15 (ISG15), IFN-induced protein with tetratricopeptide repeats 1 (IFIT1), regulated upon activation normal T-cell expressed and secreted factor (RANTES), and oligoadenylate synthetase-like protein (OASL). This study further showed that PB1 inhibited SeV-induced phosphorylation of IκBα, IRF3, and TBK1 (47). PB1 inhibition of virus-induced antiviral responses was evaluated, and overexpression of PB1 resulted in inhibition of the activation of the IFN-β promoter induced by the expression of MAVS, MDA5-N, and RIG-IN (47). Further investigations confirmed the colocalization of MAVS and PB1 inside the cytoplasm, and coimmunoprecipitation experiments showed that MAVS interacts with PB1 via its transmembrane domain. The interaction of these proteins leads to MAVS degradation in an autophagy-dependent manner, indicating that MAVS is a direct PB1 target. Moreover, this study also showed that PB1 promotes E3 ligase ring-finger protein 5 (RNF5) to catalyze K27-linked polyubiquitination of MAVS at Lys362 and Lys461. Additionally, PB1 senses and transfers ubiquitinated MAVS to autophagosomes for degradation. PB1 promotes H7N9 infection (used in this study as an IAV model) by inhibiting RIG-I/MAVS-mediated host innate immune responses (**Figure 1**, **Table 1**) by regulating the degradation cascade (47). In summary, upon IAV infection, RIG-I senses viral RNA, and hence, RLR-mediated signaling is activated. PB1 regulates the K27-linked ubiquitination of MAVS that is mediated by the E3 ligase RNF5. Furthermore, PB1 interacts with NBR1 to recognize ubiquitinated MAVS, and the PB1-RNF5-MAVS-NBR1 complex fuses with lysosomes for MAVS degradation; therefore, the MAVS-mediated innate signaling pathway is disrupted, leading to IFN-I suppression.

## PB1-F2 inhibits NF-κB signaling by binding KKβ

The PB1-F2 protein contains 90 amino acid residues that are encoded by the +1 alternate open reading frame (ORF) in the PB1 gene of some IAVs (48, 68). The influenza viruses that caused the 1918, 1957 and 1968 pandemics express the PB1-F2 protein (48). In animal models, this protein delayed the clearance of virus by killing immune cells by apoptosis, thus contributing to the pathogenesis of the virus (69). Replacement of Aspargin (N) by serine (S) (N66S) in this protein at amino acid 66 of the 1918 H5N1 IAV pandemic strain increased the virulence of this virus (70). Surprisingly, infection of mice with viruses expressing PB1-F2 with the N66S substitution led to the inhibition of ISGs at early stages of infection (71). *In vitro* assays verified the *in vivo* experimental data. Overexpression of the A/Puerto Rico/8/1934 (PR8) PB1-F2 protein in 293T cells inhibited RIG-I-regulated activation of the IFN-β reporter and the secretion of IFNs. Overexpression of the PB1-F2 N66S protein in 293T cells resulted in increased IFN inhibition compared to wild-type PB1-F2. The same results were obtained in the context of viral infection with the PB1-F2 N66S virus. Immunofluorescence assays demonstrated that both PB1-F2 66 N and 66S colocalized with MAVS, and therefore, PB1-F2 (both 66 N and 66S) was thought to inhibit IFN induction at the MAVS level, probably by direct binding (48).

Coimmunoprecipitation and yeast two-hybrid assays showed that PB1-F2 binds with IKKβ and thus suppresses downstream NF-κB signaling (**Figure 1**, **Table 1**) (49). An electrophoretic mobility shift assay demonstrated that in cells overexpressing this protein, NF-κB binding to DNA was significantly impaired and thus NF-κB signaling was suppressed; however, IKKβ kinase activity or NF-κB translocation to the nucleus was not affected. Moreover, the complete protein was indispensable for inhibiting NF-κB signaling, as neither the C-terminus nor the 57 amino acids of the N-terminus decreased NF-κB signaling (49).

As the IAV NS1 protein is a major inhibitor of IFN activation (72), this protein uses several strategies to create an anti-IAV state in virus-infected cells (73). Among the different strategies, one strategy is masking viral RNA from recognition by RIG-I (74, 75). The amino acid residues R38 and K41 at the N-terminus of NS1 are crucial for mediating the interaction with dsRNA species (74). Mutations of these residues abolished the binding between TRIM25 and NS1, a binding that is indispensable for suppressing the activation of RIG-I mediated by E3 ligase (38). Therefore, Varga et al. examined IFN activation by viruses that express TRIM25/dsRNA binding mutants of NS1 and PB1-F2 66 N or 66S (48). These findings indicated that even in the presence of NS1, which is deficient in dsRNA and TRIM25 binding, 66S, compared to 66 N, reduced the activation of IFN. Similarly, they also found that N66S, but not wild-type PB1-F2, increased the IFN inhibition of NS1 (48).

## PB2 interacts with and inhibits JAK1/STAT signaling to inhibit host innate immune signaling

The PB2 protein is important for IAV replication. The E627K substitution is responsible for replication in mammalian cells and is a dominant adaptation marker in human-adapted IAVs (51). PB2 is a negative regulator of the IFN-stimulated antiviral response and interacts with JAK1, downregulates its expression and degrades it by using proteasome machinery, thus inhibiting IFN signaling. Furthermore, the possibility of JAK1 polyubiquitination was explored by Yang et al. (51), and they found that overexpression of PB2 significantly promoted K48-linked (but not K11-, K27-, K63 linked) ubiquitination of JAK1. The amino acids Lys859 and Lys860 (K859 and K860) of JAK1 were determined to be critical for PB2 action, as substitution of these two amino acids with arginine (R) (K859R; K860R) resulted in the loss of JAK1 degradation mediated by PB2. These results indicated that the degradation of JAK1, mediated by ubiquitination at the K859/K860 residues, is important for the replication of IAVs. A past study demonstrated that in mice, 283 M/526R of PB2 from the CZ is a virulence marker for A/duck/Eastern China/JY/2014 (JY) (76). Notably, the H5 subtype of highly pathogenic AIV (HPAIV) with I283M/K526R mutations in PB2 enhanced the ability to degrade mammalian JAK1 and thus replicate with higher efficiency in mammalian cells but not in avian cells. This study provided a mechanistic explanation of the host immune evasion strategy employed by IAV that involves the degradation of JAK1 by PB2 (**Figure 1**, **Table 1**) (51).

## HA degrades IFNAR1 and IFNGR1 to facilitate IAV replication

Overexpression of IFNAR1 exerts anti-IAV effects by blocking virus replication. The HA protein activates IFNAR1 ubiquitination, thereby decreasing IFNAR1 levels and hence facilitating IAV replication inside cells. During maturation, HA is cleaved into the HA1 and HA2 subunits, and only the HA1 subunit significantly decreases IFNAR1 levels. Moreover, HA overexpression does not affect the mRNA levels of IFNAR1, and only the protein levels of IFNAR1 were decreased. The IFNAR1 levels on the cell surface affect cellular sensitivity to IFN-α/β (77–80), and HA decreases the surface IFNAR1 levels; therefore, the status of STAT1 and STAT2 was determined in the presence or absence of HA (52). HA overexpression significantly inhibited IFN-induced STAT1 and STAT2 phosphorylation (**Figure 1**, **Table 1**). IAV HA degrades IFNAR1, which helps viruses escape the host innate immune system. After this study, Xia et al. (53) thoroughly investigated the mechanism of IFNAR1 degradation by IAV HA (53). They found that, in addition to IFNAR1, HA could also degrade type II IFN (IFN-γ) receptor 1 (IFNGR1) through casein kinase 1α (CK1α). Knockdown of CK1α by small interfering RNA (siRNA) repressed the degradation of both IFNAR1 and IFNGR1 induced by IAV infection. These studies suggested that the HA protein of IAV activates the degradation of IFN receptors via CK1α, thereby facilitating viral replication (52, 53).

The molecular mechanism of IFN receptor degradation by the IAV HA protein was delineated further by Xia et al. (81), who performed mass spectrometry (MS) to identify the host protein that binds to viral HA. MS was used to identify a host protein, poly (ADP-ribose) polymerase 1 (PARP1), that interacts with the HA of IAV. PARP1 belongs to the PARP family, which regulates the differentiation and proliferation of cells (82–84). The MS result was confirmed by a coimmunoprecipitation experiment. The colocalization experiment showed that endogenous PARP1 localization was altered after IAV infection or HA overexpression. IFNAR1 expression is critical for antiviral responses mediated by IFN-I, and PARP1 promoted the replication of IAV by controlling IFNR1 levels. Knockdown of PARP1 rescued IFNAR1 levels upon HA overexpression or IAV infection, indicating the importance of PARP1 for IAV- or HA-induced reduction in IFNAR1. This mechanistic study revealed that PARP1 facilitates IAV replication by regulating HA-induced degradation of IFNR1 and thus impacts the IFN signaling pathway (81).

## PA interacts with and inhibits IRF3 nuclear translocation to suppress IFN-β activation

The polymerase acid (PA) subunit protein enhances influenza virus pathogenicity, transmission, its capability to infect a broader range of hosts, and polymerase activity and participates in restricting IFN-β production (85–88). The PA protein suppresses the production of IFN-β by interacting with IRF3, subsequently suppressing its phosphorylation, dimerization and nuclear translocation (**Figure 1**, **Table 1**). The PA protein has two functional domains, i.e., a PAN domain of 30 kDa at the N-terminal end, which contains amino acid residues 1-257, and a PAC domain of 53 kDa at the C-terminal end, which contains amino acid residues 258-716 (89). PAN conveys endonuclease activity, while PAC is a PB1 binding domain (90). The PAN domain of PA was sufficient to reduce the expression levels of IFN-β and ISG-56, similar to the complete PA protein (50), while the PAC domain failed to reduce IFN-β or ISG-56 levels (50). The coimmunoprecipitation experiments showed that the PAN domain interacts with IRF3, and thus, this fragment was responsible for suppressing the IFN-β signaling pathway. Since the PAN domain conveys endonuclease activity, a previous study demonstrated that when aspartic acid (D) was substituted with alanine (A) at amino acid 108 (D108A) in the PAN domain, the endonuclease activity of PA was abolished (91). Therefore, this mutation was explored in terms of its ability to suppress IFN-β activity (50). The D108A mutation failed to inhibit IFN-β activation, and this mutation abolished the interaction between PA and IRF3.

## Conclusions and future perspectives

A complex and dynamic interplay exists between IAVs and host innate immune responses. IAVs use various proteins that target different proteins in the host to evade the host innate immune system. During the last two decades, virus-host interactions have been thoroughly studied, and these studies provide further explanations for the mechanisms that IAVs use against the host to establish successful infection.

Several NSPs have been discovered, including PA-X, PB1-N40, PA-N155, PA-N182, and M42 NS3, whose roles in host immune evasion need to be determined (92–96). Additionally, some gaps also exist in the existing research, e.g., NS2 interacts with both IRF3 and IRF7 and inhibits the nuclear translocation of IRF7 (12); however, the mechanism of IRF3 nuclear translocation still needs to be determined. Similarly, the PB1-F2 protein colocalizes with MAVS to inhibit IFN induction (48); however, the physical interaction between PB1-F2 and MAVS still needs to be evaluated.

The conserved nature of the host innate immune system and high diversity of IAVs demands further thorough investigation to provide a deeper understanding and to enable the development of effective anti-IAV treatments that could be used against diverse IAV strains. In the future, more extensive efforts are needed to apply the current research to increase host innate immunity and control disease. As IAVs are under severe evolutionary pressure due to mutations and recombination processes, their protein characterization and functions warrant repeated thorough investigations, especially with respect to the evasion of innate host processes. Bridging the different gaps will provide directions for designing better vaccines and novel antiviral agents.

## Author contributions

FR: Writing – original draft, Writing – review & editing. ZXX: Conceptualization, Funding acquisition, Supervision, Writing – review & editing. ML: Validation, Writing – review & editing. ZQX: Validation, Writing – review & editing. SL: Validation, Writing – review & editing. LX: Validation, Writing – review & editing.
